# Understanding adaptive responses in PrEP service delivery in Belgian HIV clinics: a multiple case study using an implementation science framework

**DOI:** 10.1002/jia2.26260

**Published:** 2024-07-05

**Authors:** Jef Vanhamel, Thijs Reyniers, Bea Vuylsteke, Steven Callens, Christiana Nöstlinger, Diana Huis in ’t Veld, Chris Kenyon, Jens Van Praet, Agnes Libois, Anne Vincent, Rémy Demeester, Sophie Henrard, Peter Messiaen, Sabine D. Allard, Anke Rotsaert, Karina Kielmann

**Affiliations:** ^1^ Department of Public Health Institute of Tropical Medicine Antwerp Belgium; ^2^ Department of General Internal Medicine and Infectious Diseases Ghent University Hospital Ghent Belgium; ^3^ Department of Clinical Sciences Institute of Tropical Medicine Antwerp Belgium; ^4^ Department of Nephrology and Infectious Diseases AZ Sint‐Jan Brugge‐Oostende AV Brugge Belgium; ^5^ Department of Infectious Diseases Saint Pierre University Hospital Université Libre de Bruxelles Brussels Belgium; ^6^ Department of Internal Medicine and Infectious Diseases Cliniques Universitaires Saint‐Luc Brussels Belgium; ^7^ HIV Reference Centre University Hospital of Charleroi Charleroi Belgium; ^8^ HIV Reference Centre and Internal Medicine Erasme Hospital Université Libre de Bruxelles Brussels Belgium; ^9^ Department of Infectious Diseases and Immunity Jessa Hospital Hasselt Belgium; ^10^ Faculty of Medicine and Life Sciences LCRC Hasselt University Hasselt Belgium; ^11^ Department of Internal Medicine and Infectious Diseases Universitair Ziekenhuis Brussel Vrije Universiteit Brussel Brussels Belgium

**Keywords:** delivery of healthcare, HIV, implementation science, pre‐exposure prophylaxis, public health systems research, qualitative research

## Abstract

**Introduction:**

In Belgium, oral HIV pre‐exposure prophylaxis (PrEP) is primarily provided in specialized clinical settings. Optimal implementation of PrEP services can help to substantially reduce HIV transmission. However, insights into implementation processes, and their complex interactions with local context, are limited. This study examined factors that influence providers’ adaptive responses in the implementation of PrEP services in Belgian HIV clinics.

**Methods:**

We conducted a qualitative multiple case study on PrEP care implementation in eight HIV clinics. Thirty‐six semi‐structured interviews were conducted between January 2021 and May 2022 with a purposive sample of PrEP care providers (e.g. physicians, nurses, psychologists), supplemented by 50 hours of observations of healthcare settings and clinical interactions. Field notes from observations and verbatim interview transcripts were thematically analysed guided by a refined iteration of extended Normalisation Process Theory.

**Results:**

Implementing PrEP care in a centralized service delivery system required considerable adaptive capacity of providers to balance the increasing workload with an adequate response to PrEP users’ individual care needs. As a result, clinic structures were re‐organized to allow for more efficient PrEP care processes, compatible with other clinic‐level priorities. Providers adapted clinical and policy norms on PrEP care (e.g. related to PrEP prescribing practices and which providers can deliver PrEP services), to flexibly tailor care to individual clients’ situations. Interprofessional relationships were reconfigured in line with organizational and clinical adaptations; these included task‐shifting from physicians to nurses, leading them to become increasingly trained and specialized in PrEP care. As nurse involvement grew, they adopted a crucial role in responding to PrEP users’ non‐medical needs (e.g. providing psychosocial support). Moreover, clinicians’ growing collaboration with sexologists and psychologists, and interactions with PrEP users’ family physician, became crucial in addressing complex psychosocial needs of PrEP clients, while also alleviating the burden of care on busy HIV clinics.

**Conclusions:**

Our study in Belgian HIV clinics reveals that the implementation of PrEP care presents a complex—multifaceted—undertaking that requires substantial adaptive work to ensure seamless integration within existing health services. To optimize integration in different settings, policies and guidelines governing PrEP care implementation should allow for sufficient flexibility and tailoring according to respective local health systems.

## 1 INTRODUCTION

Oral pre‐exposure prophylaxis (PrEP) is highly effective in reducing the risk of HIV acquisition when used appropriately [1, 2]. Making PrEP broadly available and accessible is essential to reduce HIV incidence at population‐level [3, 4]. This will require service delivery models with sufficient capacity and reach to sustainably provide high‐quality PrEP services to all those who could benefit of its use [3, 5]. However, globally, implementers have grappled with finding ways to make optimal use of health system resources to scale‐up PrEP delivery [6, 7, 8]. Among other factors, differences in legal frameworks on prescription authority and available healthcare infrastructure, and low interest in PrEP among some providers, have often resulted in an intentional or “de facto” centralization of PrEP delivery through a limited number of dedicated clinics (e.g. for HIV or sexual health) [9, 10, 11].

Also in Belgium, PrEP is almost exclusively provided through specialized HIV clinics. In 2017, federal health authorities issued a set of criteria that specified conditions for PrEP to be reimbursement. Next to eligibility criteria for PrEP, this reimbursement requires PrEP to be prescribed by specialized physicians working in one of 12 certified HIV clinics (see Supporting information S1 for more details on these criteria and PrEP delivery procedures in Belgium) [12]. These clinics were originally established to provide multidisciplinary care for people living with HIV, are embedded within secondary and tertiary health facilities (mostly hospital settings), and receive separate government funding to support their HIV and PrEP programmes. In 2022, 6934 unique individuals (i.e. 59 per 100,000 population) received reimbursed PrEP at least once through a Belgian HIV clinic [13]. This constituted a 31% increase compared to 2021 and a 197% increase compared to 2018, suggesting a gradual expansion in HIV clinics’ capacity to accommodate PrEP users [13].

Successfully integrating PrEP services in existing services depends largely on the capability of frontline providers to cope with new responsibilities and additional demands (e.g. increased workload) under conditions of constraint (e.g. limited resources) [14, 15]. Previous research has shown that this integration exercise often requires adaptive changes to ensure that programmes align well with the context in which they are implemented [14, 16]. These adaptations are an inherent part of implementation processes, and require careful balancing between adherence to guidelines that protect clinical safety and ensuring a good fit with the local on‐ground realities of busy clinic environments [16]. While generic guidance on strategic opportunities for PrEP integration in existing health services exists, few studies have elucidated the role of providers and their adaptive responses in optimizing the workability of PrEP service delivery within the context of national PrEP programmes [17, 18, 19, 20]. Such insights are critical to better support and equip providers and facilities in their efforts to scale‐up PrEP.

This study aimed to investigate PrEP providers’ adaptive responses to integrate PrEP care into Belgian HIV clinics, including the challenges they faced doing so. We drew on implementation science theory to support a better understanding of more complex interactions between providers and the local implementation context across different settings.

## 2 METHODS

### 2.1 Study design

We adopted an exploratory qualitative multiple case study design, in light of the demonstrated flexibility and value of this design to study complex phenomena within their everyday context [21]. Given our interest in facility‐level implementation processes, HIV clinics constituted the cases.

### 2.2 Recruitment of study sites

As the study was of an exploratory nature, recruitment of study sites was not restricted. All 12 HIV clinics were invited to participate through the Belgian Research on HIV and AIDS Consortium (BREACH), a platform for research collaborations on HIV in Belgium. Eight clinics agreed to participate. The participating study sites accounted for geographic spread across the country, and demonstrated diversity in the annual volume of PrEP users and in type of facility (e.g. hospital‐based clinics vs. out‐patient policlinics) (see Supporting information S2).

### 2.3 Data collection

Between January 2021 and May 2022, we conducted study visits, spread over 1–4 days at each facility, to interview key informants and different types of PrEP providers, and conduct non‐participant observations.

We used the following data collection techniques:

#### 2.3.1 Key informant interviews

We interviewed key informants to get a descriptive overview of the process of design, implementation and adaptation of the PrEP programme in every case. Participants were purposively selected and included senior physicians and staff with coordinating responsibilities in PrEP service delivery. Clinic heads helped to identify and invite suitable candidates, and could also identify themselves as key informant. Additional key informants could be included through snowball sampling based on information provided by previous participants, when deemed useful by the researcher.

#### 2.3.2 Semi‐structured interviews with PrEP care providers

We interviewed a purposive sample of healthcare providers at every study site to explore their views on, and experiences with, PrEP service delivery. Potential interviewees were identified with the help of key informants, who indicated providers with crucial roles in different steps of the PrEP care process. Additional participants were recruited using snowball sampling during observations (see below).

We used open‐ended and semi‐structured interview guides, adapted according to the type of interview and provider (see Supporting information S3). Key informant and PrEP provider interviews were conducted in Dutch, French or English by a researcher with a medical background trained in qualitative research (JV), and lasted between 40 and 60 minutes. Interviews took place in‐person during the study site visit, or online (using Zoom), before or after the study site visit, according to the interviewee's preference and convenience.

#### 2.3.3 Non‐participant observation

During study visits, observations were conducted to obtain information about contextual aspects that remained unspoken or were perceived to be irrelevant for the research by interviewees intimately involved in day‐to‐day routines. Observations were guided by a semi‐structured observation guide, focused mainly on documenting the client flow through the clinic and triangulating interview data. Consistent with the iterative approach of qualitative research, data from interviews and observations could as such feed into one another [22]. For example, observations sometimes revealed particular PrEP care practices that were subsequently explored further in interviews to better understand their origins, purpose and meanings, hence contributing to a richer account. Observations also provided an opportunity to conduct informal conversations with PrEP providers (e.g. getting their immediate feedback on some observed practices), which complemented the more formal semi‐structured interviews.

Additionally, repeated informal discussions were organized between the researcher (JV) and key informants to have some “member reflections” on the ongoing process of interpretation of the data, and to further confirm or contrast observations from other study sites [23].

### 2.4 Data analysis

**Figure 1:**
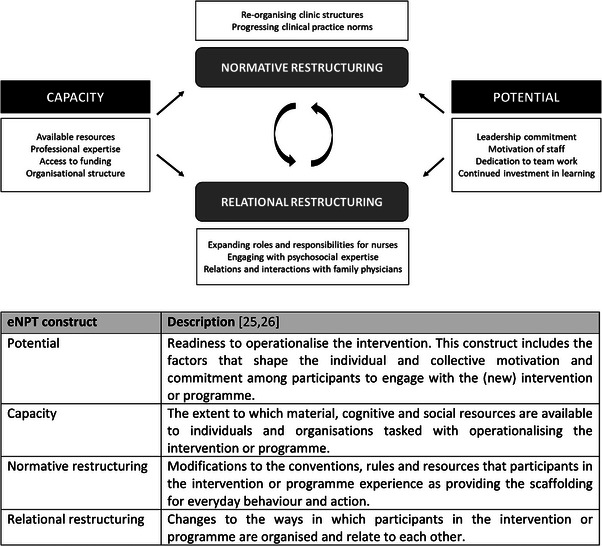
**Relation between themes identified in the qualitative data and selected eNPT constructs**. Abbreviation: eNPT, extended Normalisation Process Theory.

Interviews were audio‐recorded and transcribed verbatim. Field notes and interview transcripts were first repeatedly read through and annotated by JV, with oversight from KK. Initial impressions and observations were shared within the study team. We then used thematic analysis [24] to link inductively generated themes, derived from the qualitative data, to deductively identified constructs of extended Normalisation Process Theory (eNPT) [25, 26]. Normalisation Process Theory (NPT) is an implementation science theory originally developed to identify factors that promote or inhibit the incorporation of complex interventions in everyday practice [27, 28]. Later versions such as eNPT focused more explicitly on interactions between actors and the broader environment that may explain why implementation processes differ between settings [25, 26]. We chose this framework because it provided a good fit between our data and some of the theoretical constructs, and our specific interest in providers’ adaptive responses during PrEP implementation. We opted to engage with four particular eNPT constructs (i.e. capacity, potential, normative restructuring and relational restructuring) that were consistent with the themes identified in our data [28]. We thus used eNPT as a sensitizing tool to guide and structure deepened analysis, rather than upfront in the study design [29]. We developed and refined a coding framework through within‐case and across‐case comparison, before applying it to the entire dataset (see Supporting Information S4). Figure 1 provides a schematic overview of how we conceptualized the relationship between themes identified in the data and selected eNPT constructs. In this framework, “capacity” and “potential” refer to general contextual elements, while “normative and relational restructuring” represent mechanisms of adaptive change in relation to context. All coding activities were done in Nvivo (QSR; version 1.5).

### 2.5 Ethics statement

The research protocol was approved by the Institutional Review Board of the Institute of Tropical Medicine (ref.1431/20) and the Ethics Committee of the University Hospital of Antwerp (ref. B3002021000050). Verbal informed consent was provided by all participants, which was audio‐recorded (for interviewees) or documented in the researcher's field notes (for observations).

## 3 RESULTS

Table 1 presents an overview of data collection activities and study participant characteristics. We present our results based on the conceptual framework presented in Figure 1. We first highlight some shared characteristics in capacity and potential of HIV clinics that provide context to understand providers’ actions. We then proceed to show how interactions between capacity and potential, and processes of normative and relational restructuring, co‐determine how PrEP service delivery is implemented, referring to clinic examples. A more comprehensive and detailed breakdown of across‐clinic differences can be found in Supporting Information S5.

**Table 1:** Overview of data collection activities and study participants

Characteristic	Key informant interviews (*N* = 10)	PrEP provider interviews (*N* = 26)	Observations (time in hours)
**Setting (*N* = 8)**			
Clinic A	2	6	14
Clinic B	2	2	6
Clinic C	1	3	3
Clinic D	1	3	4
Clinic E	1	4	3
Clinic F	1	2	6
Clinic G	1	3	8
Clinic H	1	3	6
**Sex of interviewees**			n/a
Female	6	19	
Male	4	7	
**Age group of interviewees**			
Below 30	0	4	
30–50	2	15	
Over 50	8	7	
**Hierarchical position of interviewees**			
HIV clinic head	4	0	
Clinical PrEP coordinator/manager	5	0	
Other (clinical) staff	1	26	
**Professional profile of interviewees**			
Infectious disease physician	8	9	
General practitioner (attached to HIV clinic)^a^	1	2	
Nurse	0	10	
Sexologist/psychologist	0	4	
Family physician^b^	0	1	
Administrative staff	1	0	

Abbreviations: HIV, human immunodeficiency syndrome; n/a, not applicable; PrEP, pre‐exposure prophylaxis.

^a^ Refers to physicians without infectious disease specialty training who work (part‐time or full‐time) as consultant in HIV and sexual health at the HIV clinic.

^b^ Refers to a physician who worked as part‐time consultant at the HIV clinic, with a family practice outside the HIV clinic.

### 3.1 Capacity

Key informants attributed variation in PrEP care practices across settings to the absence of a streamlined—national—implementation and clinical guidance. Yet, clinicians reported benefiting from their longstanding experience in HIV and sexual health, particularly in responding to the needs of men who have sex with men (MSM), to build capacity for PrEP delivery. They described tapping into familiar professional HIV networks to acquire new information about PrEP, and compiling useful clinical guidance from published literature and through attending conferences.

Key informants across cases reported two central capacity‐related challenges underlying many adaptive changes: (1) government funding for HIV clinics was perceived to be insufficient in relation to the growing demand for PrEP, and (2) reimbursement regulations on PrEP service delivery were often deemed overly prescriptive, limiting providers’ discretion to tailor care to individual users’ needs and undertake initiatives to reduce workload for staff.

### 3.2 Potential

Clinic managers demonstrated commitment to making PrEP available because they believed PrEP was compatible with the clinic's mission and purpose. Observations revealed clinic managers often worked alongside staff as PrEP clinicians, reportedly to stay connected to on‐ground service delivery realities. Across clinics, we observed physicians and nurses making considerable investments into continuous learning about PrEP, indicative of their dedication and motivation to becoming skilled and confident PrEP providers. Providers often referred to the HIV clinic's historical focus on prioritizing HIV and sexuality‐related issues among MSM to trace their commitment and passion to providing high‐quality HIV services, including prevention, in a non‐judgemental and confidential environment.

### 3.3 Normative restructuring

#### 3.3.1 Re‐organizing clinic structures

Key informants described adaptations to PrEP services’ organizational structure as “organic” and reactive, to reduce the workload caused by a growing PrEP user cohort. Engaging additional staff (e.g. general practitioners historically working at HIV clinics and nurses) and protocolizing PrEP workflows (e.g. incorporating *pro forma* clinical checklists in electronic medical files) were common strategies to reduce logistical barriers and facilitate a team‐based approach.

Two broader organizational strategies could be delineated, partly influenced by historical differences in staffing and infrastructure: (1) clinics with already separated consultations for sexually transmitted infection (STI) care and prevention and some low‐volume clinics applied an integrated approach where providers combined PrEP services with other services at the same time (i.e. no dedicated PrEP clinics). Alternatively, in high‐volume clinics or regional hospitals with smaller teams and many competing priorities, PrEP clinics were separated from other services in terms of timing and client flow. For instance, one key informant explained how clustering PrEP visits at a particular day of the week allowed staff to better coordinate with each other and plan for follow‐up activities (e.g. notifying clients of test results), recognizing that this came with trade‐offs in flexibility for PrEP users:

*“We now have PrEP visits every Monday […] We have strongly optimised the process, meaning it is very good and efficient, but it has two disadvantages: you [clients] have to be strictly on time, and we cannot compromise on the day. […] That's the best we are able to offer.”* (physician; clinic C)

Despite clinic managers’ investments to create efficient processes, some clinics reported ongoing challenges keeping up with PrEP demand. As consultation slots were increasingly being filled‐up with clients attending regular follow‐up visits, waiting times for new clients could sometimes reach several months. One key informant in charge of clinic planning noted “*no matter how many consultation slots you foresee for PrEP, they are immediately full anyway.”* (administrative staff; clinic A).

#### 3.3.2 Progressing clinical practice norms

In the absence of national PrEP guidelines, specific PrEP care practices varied substantially across cases. Even within cases, clinicians sometimes seemingly deviated from internal protocols or policy‐issued PrEP regulations. Interviews further contextualized such practices as opportunistic ways of tailoring care to individual client needs, creating space for flexibility within increasingly structured routines. Clinicians viewed such adaptations as a natural and justifiable progression of norms based on evolving clinical experience with PrEP care. This sometimes conflicted with rigid top‐down‐issued reimbursement rules, perceived by some interviewees as relatively detached from clinical reality and sometimes even at odds with the delivery of client‐centred care. This was illustrated in the following two domains:

1. *PrEP prescribing practices*: During PrEP eligibility screening, clinicians balanced a risk‐based with a needs‐based approach, sometimes stretching the norm of appropriate PrEP indications as specified in reimbursement criteria. Refusing PrEP was experienced as difficult when confronted with specific demands of clients rooted in a need for support, wherein PrEP could present one component of a personalized management plan:
*“Some [clients] don't have an objective HIV risk, but can't experience a fulfilling sex life because an irrational fear of HIV. I don't refuse PrEP in such cases, even though it is a grey zone, but it can be part of the management, together with linking them to our sexologist for therapy.”* (physician; clinic G)
2. *PrEP care follow‐up*: Clinicians tailored the frequency, timing and location of PrEP follow‐up visits according to PrEP users’ needs and abilities to remain engaged in care. Especially for on‐demand PrEP users at low HIV risk, reducing the frequency of follow‐up visits provided a common strategy to open‐up space to receive clients waiting to start PrEP in one high‐volume clinic. In other clinics, clinicians were more hesitant to formally implement this strategy (i.e. reimbursement rules demand quarterly visits), while acknowledging the need for a more individualized approach to determine optimal follow‐up frequencies. Furthermore, providers sometimes went out of their way to accommodate PrEP users experiencing difficulties to remain in follow‐up, such as offering visits during non‐regular clinic hours or “overbooking” consultation slots, resulting in long working days. One clinic reported leveraging existing relations with infectious disease physicians in regional hospitals as a strategy to provide PrEP care to clients with poor geographical access to HIV clinics. Providers’ commitment to tailor care notwithstanding, clinic distance was still perceived a considerable barrier for some users:
  “*I once lost one [PrEP user] living in [remote village] who became HIV positive. He blamed himself, because he never came back to the clinic after having received all the information. I even prescribed PrEP to him [pause] But I think this was really an accessibility problem. He had nowhere to go [for PrEP] near him. If I had known, I would have sent him prescriptions via e‐mail.”* (physician; clinic F)

### 3.4 Relational restructuring

Across‐clinic analyses revealed how interprofessional relationships were reconfigured to successfully put organizational and flexible adaptations into practice. Normative and relational restructuring processes thus interacted to solidify new ways of working, as demonstrated in the following three practice innovations.

#### 3.4.1 Expanding roles and responsibilities for nurses

Re‐organizing clinic structures often coincided with shifting tasks and responsibilities from physicians to nurses, to manage workload more effectively. We found differences across the cases in the nature of tasks shifted to nurses, and in nurses’ degree of autonomy in PrEP care. We do not describe this diversity in detail here (see Supporting Information S5), but focus on cases with strong nurse involvement to identify crucial drivers and facilitators of this particular strategy.

1. Nurses becoming *PrEP experts*: In five clinics, nurses handled routine PrEP visits semi‐autonomously (i.e. legal frameworks do not grant them prescribing authority) with immediate physician availability in case of clinical complexities. Across‐clinic analysis revealed that clinic managers in these cases held positive attitudes towards strong nurse involvement, often influenced by positive professional experiences with nurse‐based care in a previous workplace. They had a crucial role in initiating task‐shifting movements. Important facilitators of successful nurse‐physician partnerships were: clear clinical protocols, an environment based on mutual trust and open communication across professional cadres, and physical co‐location allowing flexible supervision and training:
*“We often talk about this among the team, we say that ‘PrEP patients are not the patients of this or that doctor, but they are patients of the team’. […] and they [physicians] know we are not ‘cowboys’, that we will not do things that are beyond our competences. As soon as we have a doubt, we go knocking on their doors.”* (nurse; clinic E)
  During interviews, nurses referred to the importance of informal communication practices for their professional growth. They cited continuously exchanging knowledge and best practices among themselves, via dedicated WhatsApp groups and when meeting at conferences. Together with their increased exposure to PrEP care, these practices resulted in growing knowledge and clinical experience with PrEP, in turn fostering their professional identity of confident and proud “*PrEP experts*” committed to providing high‐quality care.
2. Nurses as *caregivers*: As nurses became more involved in PrEP care, they often took up a crucial role attending to clients’ unspoken and/or non‐medical needs. Being able to take more time than physicians, they invested in building trustful relationships with clients and provided adherence counselling and education on PrEP care tailored to clients’ competences to digest new information. Where physicians recognized being often predominantly concerned with medical care aspects amidst many competing priorities, nurses recognized such patterns and responded by providing emotional and social support, where needed:
*“I see sometimes physicians that, when they have to make choices because they have a lot on their plate, they might be inclined to spend less time on PrEP, saying these are healthy people and all goes well […] I sometimes go to the waiting room to call in PrEP clients, just to ask how they are doing. You would be surprised to hear what this brings to surface …”* (nurse; clinic A)

#### 3.4.2 Engaging with psychosocial expertise

Clinicians described increasingly experiencing challenges responding to the complex psychosocial needs of PrEP users: *“we increasingly saw people struggling with sexualised drug use or compulsive sexual behaviour where we had no clue how to guide them”* (physician; clinic G). This motivated clinic managers to work more closely with on‐site psychologists/sexologists. While interviewed sexologists reportedly valued being included in PrEP programmes because of their unique expertise, they also described their successful engagement to be dependent on frontline clinicians creating the appropriate space for clients to accept referrals.

Some clinicians recognized that the high workload did not always allow the time to adequately explore issues warranting referral, potentially resulting in missed opportunities to engage in a timely manner with psychologists/sexologists. In one low‐volume clinic, a psychologist was structurally embedded in the PrEP client flow, committed to seeing all PrEP users at least once around the time of PrEP initiation. Yet, limited funding and a fear to stigmatize the entire PrEP user population as in need of specialized psychological support were mentioned as barriers to implement similar strategies in other cases. Alternatively, in two clinics, nurses obtained an additional degree in sexology to overcome the need for referral.

#### 3.4.3 Relations and interactions with family physicians

We observed that PrEP care aspects were rarely delegated to professionals outside the HIV clinic. Yet, one clinic stood out, reporting systematically encouraging PrEP users to alternate between PrEP follow‐up visits at the HIV clinic and their family physician (FP). Interviews revealed this organizational change was driven by a high workload, and facilitated by clinic leadership supportive of the idea of including FPs in PrEP care (e.g. to increase PrEP knowledge and interest among FPs).

In other cases, relations with FPs were more passive and limited to sending written reports of consultations, or involving them only at clients’ explicit request. Clinicians had different interpretations of whether reimbursement policies allowed for FP engagement, were unsure whether clients would prefer their involvement, and/or had doubts whether FPs had the required capacity to accommodate PrEP‐related care aspects sensitive to the needs of MSM:

*“I think we should keep our specificity, and don't separate PrEP from HIV. Because with my PrEP users I am talking about sex, I am talking about drugs, STIs, HIV and also HPV, which is a complex topic in the end. […] I am not sure [FPs] know all these things plus all the other things they have to deal with in their practice.”* (physician; clinic E)

However, several clinic managers reported willingness to collaborate with interested and motivated FPs who received appropriate training to make PrEP delivery more sustainable in the long‐term.

**Figure 2:**
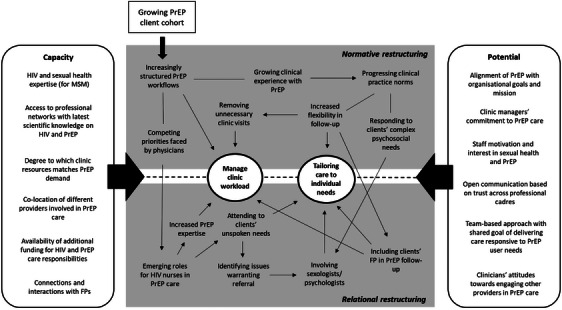
**Visual illustration of how eNPT constructs manifest and interact with each other to make PrEP care workable in Belgian HIV clinics**. Abbreviations: FP, family physician; HIV, human immunodeficiency syndrome; MSM, men who have sex with men; PrEP, pre‐exposure prophylaxis.

To conclude the results section, Figure 2 shows again the conceptual framework presented earlier (cf. Figure 1), now populated with our specific findings discussed above, providing contextualized insights into how elements of capacity and potential influence providers’ adaptive responses in Belgian HIV clinics.

## 4 DISCUSSION

This study examined how providers in Belgian HIV clinics integrate PrEP care in their routine service delivery. Using an innovative implementation theory (i.e. eNPT), we showed that implementing PrEP service delivery required considerable adaptive capacity of providers, centred around two main goals: (1) managing increased clinic workloads caused by an increasing cohort of PrEP users, and (2) flexibly respond to individual needs of PrEP users. To achieve these goals, providers had to re‐structure the normative (i.e. clinic organization and PrEP care practices) and relational environment (i.e. interactions and collaborations between health professionals) wherein PrEP service delivery was set. These adaptations and creative workarounds were largely informally implemented (i.e. not specified in local guidelines and regulations); hence, they remain largely under‐researched. We suggest, however, that they are crucial to sustain PrEP services under the conditions of broader health system dynamics and constraints.

Our findings confirm that providers’ adaptive capacity is not only absorptive (i.e. shifting resources) but also transformative, resulting in service delivery innovations [30, 31, 32]. This transformative capacity of providers was also apparent in many recent reports on COVID‐19‐induced adaptations to PrEP service delivery [33, 34, 35, 36]. Yet, considerably fewer studies have focused on PrEP providers’ adaptive responses under “normal” conditions [19, 20]. We showed that PrEP providers challenged established norms on PrEP care, leading to adaptations to the policy‐intended model of specialist physician‐led and HIV clinic‐based quarterly visits. Such adaptations distributed workload more effectively, and simplified service delivery for users. Most notable was the move in several clinics to more nurse‐led PrEP service delivery. Previous studies have reported on nurse‐led PrEP delivery as a way to increase service capacity without additional resources [37, 38]. We provide further insights into factors that can drive (e.g. clinic managers advocating for a nurse‐led approach) and enable (e.g. informal communication channels, co‐location with supervising physicians and continuous “on‐the‐job” training) nurse involvement in PrEP. Moreover, we demonstrate the value of nurse‐led PrEP delivery beyond expanding capacity, showing how nurses delivered essential contributions to the quality of care (e.g. offering non‐medical support and tailoring care to individuals’ unique circumstances).

The application of eNPT provided a useful theory‐informed approach to examine under‐recognized health system aspects of PrEP care implementation, by putting the focus on inter‐relations between providers, PrEP care and the larger service delivery context [39]. In particular, our study highlights the crucial role of providers’ commitment, values and interactions—also referred to as systems “software” [40, 41]—as essential enablers of effective service integration [42, 43, 44]. We identified many of these software factors under the eNPT construct of “potential.” This suggests that provider‐related challenges with PrEP implementation in specialized clinics have less to do with capability issues that fall within individual providers’ control (e.g. relevant expertise, inherent motivation and ability to innovate), than with the constraints associated with rigid systems. Through the lens of eNPT, many of the observed adaptive responses (i.e. the normative and relational restructuring) can then be viewed as providers’ attempts to introduce flexibility and compensate for structural restrictions in PrEP service delivery systems. For instance, the reported high workload among providers and geographical accessibility issues among clients reflect the undesired consequences of a national PrEP delivery system with limited access points. Using their individual and collective agency, PrEP providers extended policy‐issued regulations on PrEP care to reduce pressure on HIV clinics (e.g. reducing visit frequency and involving FPs), and realize their value‐driven ambition to provide client‐centred care. Such processes of bottom‐up “tinkering” with top‐down‐issued rules and regulations have previously been shown essential to bridge the gap between practice and policy, preserving quality of care, but have not received much recognition in PrEP implementation research [32, 45, 46]. Yet, a recent global consultation of PrEP providers suggests that tensions between flexible practices and standardized guidelines are common and constantly negotiated [47].

Importantly, the potential and capacity of providers to adapt and overcome systemic constraints is not without limits, and often requires them to do more with less while juggling many competing priorities (e.g. HIV care for an ageing population, rising STI rates and emerging infectious diseases like COVID‐19 and mpox) in a context of limited funding for HIV and sexual health services [48, 49]. There are indications that these systemic constraints may have negative effects on client outcomes. For instance, capacity constraints with PrEP delivery through sexual health clinics in the United Kingdom and The Netherlands have been reported to restrict timely access to PrEP, with occasionally long waiting lists and sporadic reports of individuals who acquired HIV while on the waiting list for PrEP [50, 51]. Moreover, a recent mixed‐methods study among Belgian PrEP users described how limited client‐provider interactions in HIV clinics could result in missed opportunities to address clients’ psychosocial needs [52]. Our findings concur with insights from a qualitative study among providers in Zambian HIV clinics showing that such suboptimal client experiences should not be understood as a lack of provider awareness or commitment to client‐centred care [53]. Indeed, providers in our study accurately identified the need to adapt care practices to better meet PrEP users’ psychosocial needs, but experienced organizational constraints to implement a more comprehensive PrEP service (e.g. staff shortage and lack of funding).

Our findings have practical implications to further optimize PrEP scale‐up. First, policymakers should create conditions that promote providers’ adaptive capacity and innovative potential, and give clinic managers and staff the autonomy to effectively adapt care processes to local circumstances [46]. Financial and regulatory frameworks governing PrEP rollout should ensure sufficient resources to organize multidisciplinary care in specialized clinics according to local PrEP demand, while legitimizing different practice‐based models of PrEP delivery. This may include granting PrEP prescription authority to specialized nurses and incentivizing the participation of primary care providers, such as FPs and pharmacists, in PrEP care [54, 55, 56]. Such initiatives will distribute the workload more evenly across health system actors, improve (timely) access to PrEP and ensure more client‐centred care. In particular, previous research in Belgium shows that FPs are willing to be more involved in PrEP care, and that about half of the PrEP users would accept follow‐up visits with their FP [52, 57]. While we observed the emergence of informal collaborative care models between specialized services and FPs in at least one HIV clinic, other clinics were waiting for such models to be first legitimized by reimbursement regulations before implementing them. Lastly, a common platform is needed for practitioners to exchange and learn from best practices in PrEP service delivery across settings in real‐time, and work towards a streamlined clinical and implementation guidance. This guidance should be regularly updated, and indicate a set of minimum PrEP care standards while allowing for sufficient flexibility to accommodate facility‐level variation in service delivery capacity.

### 4.1 Strengths and limitations

This study draws on a rich dataset that included multiple data sources across eight distinct clinical settings. In addition, the triangulation of data collection methods (i.e. interviews and observations), including iterative consultation with key informants, contributed to the trustworthiness of the obtained data. The eNPT provided a valuable tool to explain adaptive change as resulting from complex interactions between context and providers, and needed to sustain PrEP care implementation in a real‐world health system. In this case, the classical distinction made between context and intervention (i.e. PrEP service delivery) is blurred; both are closely interconnected and continuously mediated by implementors’ actions in an uncertain and rapidly changing environment. The potential to account for the emergent and dynamic role of context, and the agency implementors exert over it, is a specific advantage eNPT offers over determinant frameworks (e.g. Consolidated Framework for Implementation research) that allow for a comprehensive but rather static description of context [58].

Our analysis relies greatly on providers’ self‐reporting, and this may have led to underreporting of practices deemed illegitimate or substandard by informants. We tried to mitigate this by having interviews conducted by an experienced qualitative researcher, and triangulating interview data with observations. There was a possible interpretation bias from the lead researcher (JV) due to his outsider position and/or specific interests, which we tried to counter by having semi‐structured interview guides and involving key informants in follow‐up discussions and manuscript review. Although COVID‐19‐induced adaptations were retrospectively identified (e.g. offering tele‐visits during the lockdown period), these were generally not sustained at the time of the study, and we did not explore them in great detail.

## 5 CONCLUSIONS

This study showed that effective PrEP care implementation in Belgian HIV clinics constitutes more than executing a standardized set of activities according to prescribed protocols or policies. The use of eNPT as a guiding theoretical framework allowed us to understand effective PrEP implementation as an evolving process shaped by the dynamic contributions of providers, which are in turn influenced and constrained by broader health system factors. Our findings suggest that scaling‐up PrEP services to maximize impact on the HIV epidemic will require organizational policies and systems that align with the adaptive and innovative capacity of providers to differentiate and simplify PrEP services.

## COMPETING INTERESTS

The institution of TR has received fees from ViiV/GSK for attending advisory meetings. Other authors do not report any competing interests.

## AUTHORS’ CONTRIBUTIONS

Study conceptualization: JV, TR, BV and CN. Development of data collection tools: JV, TR, BV, CN and AR. Data collection: JV. Data analysis: JV and KK. Interpretation of data: TR, BV, SC, CK, DHV, JVP, AL, AV, RD, SH, PM and SDA. Writing the first draft of the manuscript: JV. Review and writing of the final manuscript: all authors. All authors have read and approved the final manuscript.

## FUNDING

This study was funded by the Flemish Research Foundation as part of a Strategic Basic Research project (FWO‐SBO) (grant number: S004919N). The funder had no role in the study design, data collection and analysis, and manuscript review.

## CME STATEMENT

This article is published as part of a supplement supported by unrestricted educational grant by ViiV Healthcare.

Credits Available for this Activity: American Medical Association (AMA Credit).

Washington University School of Medicine in St. Louis designates this enduring material for a maximum of 1 AMA PRA Category 1 Credit™. Physicians should claim only the credit commensurate with the extent of their participation in the activity.

## Supporting information


**File S1**: Belgian policy on the conditional reimbursement of oral PrEP for HIV prevention at the time of the study period

**File S2**: Background on the different study sites included

**File S3**: Interview and observation guide used for qualitative data collection

**File S4**: Extended Normalisation Process Theory constructs, definitions, themes and sub‐themes derived from the data

**File S5**: Detailed overview of the variation in PrEP service delivery across the cases

## Data Availability

The entire qualitative datasets presented in this article are not readily publicly available because they contain information that could compromise the privacy of our research participants. Additional data are available from the first author on reasonable request.
