# Supplementary material for: Understanding adaptive responses in PrEP service delivery in Belgian HIV clinics: a multiple case study using an implementation science framework
Source: J Int AIDS Soc. 2024 Jul 5;27(Suppl 1):e26260. doi: 10.1002/jia2.26260 (PMC11224588; doi:10.1002/jia2.26260)
Supplement: Supplementary file 1 — File S1: Belgian policy on the conditional reimbursement of oral PrEP for HIV prevention at the time of the study period [file JIA2-27-e26260-s004.docx]

**Understanding adaptive responses in PrEP service delivery in Belgian HIV clinics: a multiple case study using an implementation science framework**

**Supplementary file 1.**

**Belgian policy on the conditional reimbursement of oral PrEP for HIV prevention at the time of the study period (adapted from the Ministerial Decree on PrEP; June 2017).**

| 1. **To be considered eligible for PrEP reimbursement, individuals should have a confirmed HIV-negative status and meet at least one of the following criteria:** | |
| --- | --- |
| **Men who have sex with men** | **Individuals at high risk of HIV** |
| Having engaged in condomless anal sex with at least two different partners in the last 6 months. | People who inject drugs and share needles. |
| Having had more than one STI (syphilis, chlamydia, gonorrhoea or primary hepatitis B or C infection) during the last 12 months. | People exposed to condomless sex while engaging in sex work. |
| Having used post-exposure prophylaxis (PEP) at least two times in the past 12 months. | People who are exposed to condomless sex with a high risk of HIV (e.g. having sex partners in countries with high HIV prevalence). |
| Having used psycho-active drugs during sex (‘chemsex’). | Partners of people living with HIV who are not virally suppressed. |
| 1. **The reimbursement is granted on a yearly base if PrEP was prescribed by a physician who meets all of the following criteria:** | |
| The specialist physician is affiliated to a certified HIV clinic (i.e. HIV Reference Centre), commits to participate in registering data for national surveillance and organising 3-monthly follow-up visits for PrEP, including performing the recommended laboratory testing (syphilis, chlamydia, gonorrhoea or primary hepatitis B or C infection). | |

**Abbreviations:** HIV, human immunodeficiency virus; PrEP, pre-exposure prophylaxis; STI, sexually transmitted infection.

**PrEP service delivery procedures.**

PrEP care in HRCs typically entails an initiation visit with eligibility screening, assessing signs and symptoms of an acute HIV or other sexually transmitted infection (STI), providing information on different PrEP regimens (i.e. daily and on-demand PrEP) and conducting HIV, STI and other relevant laboratory tests (e.g. creatinine for kidney function). After PrEP initiation, quarterly follow-up visits are organised, involving HIV testing, STI screening, re-evaluating the need for PrEP, delivering 3-month PrEP prescriptions and providing counselling in adherence and sexual health, as needed.
