# Supplementary material for: Understanding adaptive responses in PrEP service delivery in Belgian HIV clinics: a multiple case study using an implementation science framework
Source: J Int AIDS Soc. 2024 Jul 5;27(Suppl 1):e26260. doi: 10.1002/jia2.26260 (PMC11224588; doi:10.1002/jia2.26260)
Supplement: Supplementary file 2 — File S2: Background on the different study sites included [file JIA2-27-e26260-s001.docx]

**Understanding adaptive responses in PrEP service delivery in Belgian HIV clinics: a multiple case study using an implementation science framework**

**Supplementary file 2.**

**Background on the different study sites included.**

**Table 1. Overview of key characteristics of the different clinics at the time of the study period.**

**Abbreviations:** FP, family physician; GP, general practitioner; n/a, not available; PrEP, pre-exposure prophylaxis.

† Routine involvement refers to clinicians that every client encounters during either a PrEP initiation or follow-up visit, and excludes providers only encountered on a referral basis.

§ Refers to physicians without infectious disease specialty training who work (part-time or full-time) as consultant in HIV and sexual health at the HRC.

$ Refers to physicians specialised in family medicine, and who work outside the HRC in a dedicated family practice.

‡ Data from national routine surveillance reporting on PrEP, coordinated by Sciensano, Belgium. Data are self-reported by the different HIV Reference Centres.

| **Study site characteristic** | **Clinic A** | **Clinic B** | **Clinic C** | **Clinic D** | **Clinic E** | **Clinic F** | **Clinic G** | **Clinic H** |
| --- | --- | --- | --- | --- | --- | --- | --- | --- |
| **Facility type** | National referral policlinic | Academic referral hospital | Regional referral hospital | Regional referral hospital | Academic referral hospital | Academic referral hospital | Academic referral hospital | Academic referral hospital |
| **Profile of clinicians routinely involved in PrEP service delivery^†^** | Infectious disease specialists; GPs^§^; nurses | Infectious disease specialists; GPs^§^; nurses | Infectious disease specialists; nurses | Infectious disease specialists; nurses | Infectious disease specialists; nurses | GPs^§^; nurses | Infectious disease specialists; nurses; psychologist | Infectious disease specialists; nurses; FPs^$^ |
| **Yearly number of PrEP users with at least one PrEP consultation**^‡^ |  | | | | | | | |
| 2019 | 1099 | 912 | 208 | 177 | n/a | 200 | 226 | 527 |
| 2020 | 1341 | 1032 | 243 | 172 | 367 | 220 | 167 | 637 |
| 2021 | 1537 | 1298 | 334 | 232 | 462 | 318 | 219 | 771 |
| **Yearly number of new PrEP starters**^‡^ |  | | | | | | | |
| 2019 | 426 | 632 | 90 | 61 | n/a | 110 | 89 | 218 |
| 2020 | 372 | 572 | 67 | 36 | 123 | 88 | 51 | 188 |
| 2021 | 397 | 724 | 92 | 60 | 177 | 115 | 61 | 247 |
