# Supplementary material for: Understanding adaptive responses in PrEP service delivery in Belgian HIV clinics: a multiple case study using an implementation science framework
Source: J Int AIDS Soc. 2024 Jul 5;27(Suppl 1):e26260. doi: 10.1002/jia2.26260 (PMC11224588; doi:10.1002/jia2.26260)
Supplement: Supplementary file 3 — File S3: Interview and observation guide used for qualitative data collection [file JIA2-27-e26260-s005.docx]

**Understanding adaptive responses in PrEP service delivery in Belgian HIV clinics: a multiple case study using an implementation science framework**

**Supplementary file 3.**

**Key informant interview guide.**

**PrEP provider interview guide.**

**Observation guide.**

1. **Key informant interview guide.**
2. **Respondent background**

- ***Can you tell me something about yourself in terms of educational background, professional career and current activities you perform as part of your job?***
- ***How would you describe your role in the HIV Reference Center? And can you tell me some more about the work you do around PrEP?***

1. **Study site characteristics**

- ***How would you describe the integration of HIV prevention services into the working of the HRCs?***
  - What is the role of the HRC in HIV prevention and sexual health?
  - What services are available for HIV prevention and sexual health?

1. **PrEP programme design and introduction**

- ***Can you tell me when and how PrEP was introduced at the HRC?***
- Which events led to the introduction of PrEP? And when was this?
- Was there any guidance during this process of introducing PrEP at the HRC? Where did you get information on how to get organized, and what services to provide?
- Looking back on it, was this an easy process?

***- What was done to prepare and follow-up the implementation of PrEP delivery at the HRC?***

- Were there any clinical guidelines available?
- How were clinical guidelines or protocols, if any, adopted within the team?
- How were decisions made about which type of providers would be involved in PrEP care?
- Which changes were made to the team (1), the guidelines (2), and the organization of care (3) over time?

1. **Operational aspects about PrEP service delivery**

- ***How was PrEP delivery integrated in the routine services that were already available at the HRC?***
- How were tasks divided within the team?
- How did you deal with the additional workload caused by PrEP?
- Was the same appointment system implemented for PrEP compared to other services at the HRC?
- What did you find challenging with regards to integrating PrEP services into the existing workflow?
- ***Supposed that I am interested in taking PrEP, or I am referred to the HRC by another physician, can you walk me through a typical first PrEP visit at the HRC?***
- Who does what at every stage?
- Which guidelines are used for that? (*collect later*)
- *For specific practices*: How is that being done (e.g. eligibility screening)? Can you given an example of how you would do that?
- Can clients get PrEP on the same day? Why (not)?
- Is this workflow according to a protocol? (*collect later*)
- Who schedules the next appointment? How much time is there in general between visits?
- Is there a difference between a PrEP initiation visit and subsequent visits?
- ***How is follow-up care for PrEP clients arranged, both with providers within and outside the HRC?***
- How would you describe collaborations with other health professionals, if any?
- Which information is shared with clients’ family physicians, if they have one, and how?
- What do you find challenging, if anything at all, in responding to PrEP clients’ care needs?
- How do you respond to any needs of PrEP clients related to
  - Mental health?
  - Substance abuse (incl. chemsex)
  - STIs

1. **Monitoring and evaluation**

- ***How is the monitoring and evaluation of the PrEP programme organised?***
- Are there any data related to PrEP users or the programme that are collected systematically? If yes, can you explain how this is done?
- Who is responsible for this?
- How are the necessary data retrieved?
- Are these activities streamlined with other HRCs?
- ***Is there something like a ‘PrEP team’ in the HRC that acts as an overarching body to watch over the program’s day-to-day functioning or that takes the lead on PrEP care?***
- Why (not)?
- Is there another way you keep up-to-date with on-ground service delivery issues or evolutions? How does that work?
- *If there is a team:* Which providers or staff are part of this ‘team’?
- *If there is a team:* What activities does this team perform?
  - Is training part of it?
  - Is updating clinical guidelines part of it?
  - Is discussing/reviewing clinical practices part of it?
  - Anything else?

1. **PrEP provider interview guide.**
2. **Respondent background**

- ***Can you tell me something about yourself in terms of educational background, professional career and current activities you perform as part of your job?***
- ***How would you describe your role in the HIV Reference Center? And can you tell me some more about the work you do around PrEP?***

1. **The PrEP care process and notions of required skills and expertise**

*PrEP is a relatively new intervention, and we are still learning how to best provide it. I would like to take some time to go through your experience with providing care for PrEP clients, and your thoughts on how care is organized here at the HRC. Is that okay with you? [confirmation from participant] Great, so let me start with getting a bit of a clearer picture of what happens exactly at a PrEP consultation here at the HRC.*

- ***Imagine that I am a first-time client coming to your consultation for PrEP, can you walk me through what would happen? How would you usually handle such a first-time visit?***
  - When would I first come into contact with you?
  - Where are you located? (e.g. separate cabinet or desk? Proximity to other providers?)
  - What do you do during a typical PrEP visit (focus on first-time visit)?
    - Eligibility screening
    - Education
    - Counseling
    - Lab testing
    - PrEP prescription
    - Vaccinations
  - Which other providers would I meet before or after visiting you? What would they still do on top of what you do?
  - How is this different or similar from follow-up visits (e.g. would I meet you again every time?)
- ***You mentioned to me different care aspects that are part of a typical PrEP visit. Let me go over each and one of them, and get some more insight of what they all require of you.***
  - *Assessing who are appropriate PrEP candidates*: how do you do this? Are there certain criteria you apply? Do you follow the reimbursement criteria? Why (not)?
  - *Providing education on PrEP* : Dou you have a standard way of going about this? What skills would you say it takes to provide good education on PrEP? What helps you to provide good information (e.g. are there any materials you use to support your message?). Would you tailor this information according to the type of client in front of you? In what way?
  - *Providing counseling on sexual health*: Apart from PrEP as such, do you engage in a conversation on sexual health in general (e.g. risk of other STIs, condom use, other prevention available options, family planning etc.)? Why (not)? Some providers might feel uncomfortable talking about sex, can you relate to that? Are there any materials you use to support your message?
  - *Prescribing laboratory tests*: Who decides which laboratory tests are done at a PrEP visit? What is this based on (e.g. guidelines, client profile or history, consensus in the team etc.)? Who can prescribe the necessary laboratory testing?
  - *Prescribing PrEP*: Who delivers the prescription for PrEP? How much PrEP is prescribed at each time (e.g. multi-month dispensing)? Does this sometimes vary? If yes, when? Who decides this?
  - *Providing follow-up care:* What does follow-up for PrEP clients entail once they have started on a PrEP regimen? How are you involved in this? Does the frequency and nature of follow-up sometimes vary between individuals? Can you give some examples of how and when it can be different?
  - *Responding to other sexual an mental health needs*: Are you sometimes confronted with other sexual and mental health needs of clients? How do you deal with this? What skills or expertise do you require to meet needs and demands from clients in this regard?
- ***As you might have noticed, I am interested in trying to describe what it takes to “do PrEP well”, in terms of skills, expertise, values and practices. Can you name three things (e.g. specific skills, attitudes or values) that, in your experience, are vital to ensure ‘good quality care’ for PrEP?***
  - Why the choice for these three elements? How would you rank them according to importance
  - How do you implement these three things in your day-to-day practice?
  - What has supported you, or still supports you, to provide good quality care for PrEP clients?
  - What barriers do you sometimes encounter to live up to putting these three things into practice? Can you give examples?
  - What does it take for one to acquire this expertise?
  - How does this expertise relate to the following factors (*probe for each*):
    - professional training
    - personal norms and values
    - being part of a multidisciplinary team
    - access to certain resources (e.g. equipment, guidelines or infrastructure)
    - experience (years in the field ; engaging regularly in sexual health care)
    - being part of a key population for PrEP/knowing the key populations well
    - other?

1. **Client-centered care and shared decision making**

*In the provision of health care, the concept of “patient-centered” or “client-centered” services has been receiving more and more attention. This approach means “providing care that is respectful of, and responsive to, individual patient preferences, needs and values, and ensuring that patient values guide all clinical decisions”.*

- ***When you think again about – let’s say – the last five to ten clients you attended to for PrEP, how would you say you were able to implement ‘client-centered care’ ?***
  - (*to stimulate thinking*) Would you say you treat everybody the same?
  - How were you able to identify and respond to specific clients’ needs?
  - How were you able to identify and respond to clients’ specific preferences?
- ***One aspect of “client-centered care” is shared decision making between the provider and client. To what extent do you engage clients themselves in decisions for each of the following care aspects:***
  - *Whether or not PrEP is a good option for clients*: How do you support clients in this decision? Would you sometimes advise against PrEP? When and why? Would you sometimes advise alternative HIV prevention options instead of PrEP? When and why?
  - *Which PrEP regimen to start (daily or event-driven)*: How do you support clients in this decision? Would you sometimes advise against certain regimens? When and why?
  - *Which laboratory tests to perform*: To what extent can clients choose which tests are performed as part of screening or follow-up (incl. biochemistry, organ functions, HIV and STI testing)? Which tests can be more flexible than others? Are the same tests always performed in every individual? Why (not)?
  - *Which vaccines to get (incl. HPV)*: How are vaccinations discussed with clients? Would you recommend an HPV vaccine to all individuals? Why (not)?
  - *Plans around the practice of ‘safe sex’ (e.g. condom use)*: Do what extent is a strategy for protection of other STIs (besides HIV) being discussed with all clients? How would you describe your responsibility versus the responsibility of clients when it comes to sexual health promotion and prevention?
  - *On timing (date and time) of follow-up visits (for testing and consultation)*: To what extent do clients have a choice when to return for a follow-up visit (frequency and timing during the week) ? Can clients visit the same provider every time? How important would you think it is to have the same provider attending to clients?
  - *On how to involve clients’ GP in PrEP care*: To what extent do you discuss with every new client how they would like their GP to be involved in PrEP care? If yes, why is it important to do this? If not, why is it not always discussed?
- ***In your experience, what can be barriers to practice ‘shared decision making’ in PrEP care.***
  - When is it not desirable? Why?
  - When is it not feasible? Why?
  - Which factors can make it difficult to practice ‘shared decision making’
    - probe for policy, health systems, facility-related and provider-related factors
- ***In your experience, what are crucial facilitators to practice ‘shared decision making’ in PrEP care?***
  - Which practices or interventions stimulate clients’ engagement?
  - How can the working of the HRC be made more conducive to practicing ‘shared decision making’ ?

1. **Reflections on the service delivery model for PrEP**

- ***Based on your experience, what would you say are three key advantages of providing PrEP through a specialized setting such as the HRC? Why do you think that is?***
  - What are some disadvantages of providing PrEP through the HRC? Why do you think that?
  - Around the world, people are thinking of what good service delivery models for PrEP could look like. There is increasing attention for the role of nurses on the one hand, and first-line health workers, such as family physicians, on the other hand. From your experience, what could be the added value of involving clients’ family physician in PrEP care?
  - Alternatively, according to you, what conditions would be required for family physicians to be more involved in the delivery of PrEP? Why is that?
  - Do you feel clients could benefit of more collaboration between the HRC and their family physician? If yes, how could this collaboration look like? If no, why is that?
  - Another strategy I have observed in different HRCs, is task-shifting and task-sharing with nurses. In what way does this strategy benefit clients? And in what way does it benefit the providers at the HRC?
- ***To what extent do you feel that PrEP services at the HRC are a good fit for all clients who could benefit of its use? Why is that so?***
  - Differentiate between:
    - Geographical access (distance to the facility)
    - Acceptability (e.g. opening hours, waiting times)
    - Affordability (cost & reimbursement regulations)
    - Accommodation (how setting is constructed, “LGBTQ-friendliness”, ensuring a ‘safe’ and inclusive environment)
    - Availability (sufficient staff and resources to attend timely to clients)
  - Are there any groups that are insufficiently reached by the PrEP services at the HRC? If yes, which ones and why do you think that is?
  - In your opinion, what could be done to reach those groups better? For instance, are there other providers outside the HRC that could be involved?

1. **Observation guide**

| **Document type** | **Observation guide** |
| --- | --- |
| **Study Site** |  |
| **Name observer** |  |
| **Observation time span**  **(total duration in mins.)** |  |
| **Content** | 1. **Setting** 2. **People** 3. **Interactions (incl. clinical care)** 4. **Client flows** |

**DESCRIBING THE SETTING**

***A. Description of the physical infrastructure and location of the HRC***

- ***Where is the HRC located? (city/village, city centre vs. out of town)***
- ***Is the HRC easy to find/navigate?***
- ***Is the HRC easy to reach by public transport?***
- ***Is the HRC located in a separate building, or rather embedded in a larger clinic/hospital setting?***
- ***What are the opening hours of the HRC?***
- ***To what extent is the HRC setting different from a “standard" clinical setting?***
- ***What are differences/similarities with previously visited HRCs?***

***B. If possible, include pictures of the HRC, or clinic wherein the HRC is embedded***

***C. Description of the physical setting where PrEP services are provided***

- ***Criteria to differentiate settings***
  - ***Extent to which a setting is ‘PrEP-specific’ (tailored to PrEP clients)***
  - ***Secluded space vs. open (e.g. for privacy)***
  - ***Strictly organized vs. loose***
- ***Description of the registration desk/room***
  - ***Where do PrEP clients register? (far from clinic entrance? Which department or unit in the clinic/hospital?)***
  - ***Is there a queue or waiting area for registration?***
  - ***Other privacy-related aspects (e.g. approaching patients, visible files, etc.)***
  - ***For which other services can clients register at the same desk? (e.g. HIV services, infectious diseases, general internal medicine, travellers, etc.)***
  - ***Is there health promotion/prevention material available at the registration desk?***
  - ***Are condoms or any other protection methods available at the registration desk?***
  - ***Do PrEP clients mix with non-PrEP clients after they have registered?***
- ***Description of the waiting room***
  - ***Is the waiting room close to the registration desk?***
  - ***How is the waiting room located in relation to other corridors/rooms?***
  - ***Is it noisy? Are many people passing by?***
  - ***Is there enough space to sit and to keep distance***
  - ***Is the room crowded?***
  - ***How are people positioned towards each other?***
  - ***Are there health promotion messages on the walls?***
  - ***Are there PrEP messages on the walls?***
  - ***Are there PrEP information folders available to read?***
  - ***Is there exposure to any other sexual health messages in the waiting room?***
  - ***Are condoms or any other protection methods available in the waiting room?***
  - ***When do (PrEP) clients leave the room? Does it take a long time?***
  - ***Who calls them out, and in what way (by name and surname?)***
- ***Description of the consultation room***
  - ***Where is the consultation room located?***
  - ***Does the set-up ensure privacy is protected (window, doors, sound-proof etc.)?***
  - ***Who is present in the consultation room?***
  - ***How are client and provider positioned towards each other?***
  - ***Is the room decorated with (health) messages/posters?***
  - ***How big is the room?***
  - ***What else makes up the setting of the consultation room (examination table, examination tools, posters/books, etc.)***
  - ***Are condoms available in the consultation room?***
- ***Description of other room***
  - ***Laboratory/sampling area (who is there, set-up, privacy, possibility to lie down when drawing blood samples, possibility of self-sampling, tools & instructions for self-sampling, etc.***
  - ***Other areas?***
- ***How and where do clients exit?***

***D. If possible, include pictures of the different areas that are part of PrEP services***

| ***Drawing of a map of the HRC & setting where PrEP care is delivered*** |
| --- |

1. **PERSONS**

***A. Clients visiting the HRC***

- ***Describe the entrance of the HRC clinic/hospital***
  - ***Is it crowded?***
  - ***Are there queues of people?***
  - ***What profile of clients are there?***
    - ***Age groups? Gender(s)? outspoken sexuality?***
    - ***Couples or groups of people?***
    - ***Idea of cultural and ethnic diversities?***
    - ***What languages are spoken?***

***B. Registration for PrEP services***

- ***How many people are there at the registration desk?***
- ***Are clients coming in alone/in pairs/with multiple persons together?***
- ***What profile of clients***
  - ***Men vs women***
  - ***Age groups***
  - ***Cultural and ethnic diversity***
  - ***Couples? (man-man, man-woman, woman-woman)***
  - ***Particular things about***
    - ***Dress code***
    - ***Body language***
    - ***Posture, body art, hair style***
    - ***…***
- ***Who is welcoming the clients?***
  - ***How many clerks are there?***
  - ***Man vs. woman?***
  - ***Wearing a uniform?***

***C. Waiting room***

- ***Who is present in the waiting room?***
  - ***Profile of clients in the waiting room***
    - ***Men vs women***
    - ***Age groups***
    - ***Cultural and ethnic diversity***
    - ***Couples? (man-man, man-woman, woman-woman)***
    - ***Particular things about***
      - ***Dress code***
      - ***Body language***
      - ***Posture, body art, hair style***
      - ***…***
- ***Description of people passing by?***

***D. Consultation room***

- ***Who is present in the consultation room? (type of health care provider, client – how many at the same time?)***
- ***How is the health care provider dressed (e.g. coat, uniform, name tag with type of professional mentioned,…)***
- ***Profile of clients in the consultation room***
  - - ***Men vs women***
    - ***Age groups***
    - ***Cultural and ethnic diversity***
    - ***Couples? (man-man, man-woman, woman-woman)***
    - ***Particular things about***
      - ***Dress code***
      - ***Body language***
      - ***Posture, body art, hair style***
      - ***…***

***E. Other rooms & spaces***

- ***What other providers are present?***
- ***How are different providers dressed?***
- ***Are different health care providers recognizable as such (different uniforms, badges etc.)***

1. **INTERACTIONS**

***A. Registration desk***

- ***How are clients interacting with each other?***
  - ***Non-interaction?***
  - ***Waiting in line?***
  - ***Talking to each other?***
  - ***Non-verbal cues (e.g. of impatience) ?***
- ***How are clients interacting with health personnel?***
  - ***How do they greet each other?***
  - ***Attitude of client and clerk***
  - ***Open conversation vs. closed (or distant)***
  - ***body language of client and clerk***
  - ***What information is given?***
    - ***About where to go?***
    - ***About estimated time clients will have to wait?***
    - ***About what is going to happen from there?***
    - ***Is there an information leaflet or folder handed over?***
    - ***Are clients given a form they need to fill out ahead of the consultation?***

***B. Waiting room***

- ***How are clients behaving?***
  - ***Reading?***
  - ***Filling out a form?***
  - ***Listening to music?***
  - ***Other?***
    - ***Non-verbal communication? (sighing, smiling, ignoring, looking bored,…)***
- ***How are clients interacting?***
  - ***Non-interaction?***
  - ***Interaction between clients who came together?***
  - ***Interaction between ‘strangers’?***
- ***How are clients interacting with other health personnel?***
  - ***Are clients reaching out to personnel with questions, concerns etc. (e.g. waiting period is too long)***
  - ***How are clients called out for their appointment?***
  - ***How do clients react when called out for appointment?***

***C. Provider-client interaction***

| ***Date :***  ***Provider profile :***  ***Type of contact (e.g. clinical consultation, counseling, lab, etc.) :*** | |
| --- | --- |
| ***Type of interaction*** | ***Notes from observation*** |
| ***Greeting between the client and provider.*** |  |
| ***Exploration of the reason of the visit.*** |  |
| ***Active listening from the provider.*** |  |
| ***A demand for PrEP is identified.*** |  |
| ***Exploration of motivation and reasons to take PrEP.*** |  |
| ***Exploration of safe sex practices.*** |  |
| ***Sexual history taking.*** |  |
| ***Assessing PrEP eligibility.*** | **Using tools for this? Based on what?** |
| ***Exploration of the possibility of a recent HIV exposure.*** |  |
| ***Exploration of substance abuse (alcohol, drugs, chemsex, etc.).*** |  |
| ***Exploration of possible mental health problems/concerns.*** |  |
| ***Introduction of PrEP (what is it, efficacy, possible side effects).*** |  |
| ***Difference between daily and event-driven (or non-daily) PrEP.*** |  |
| ***Discussion on how to safely start and stop PrEP.*** |  |
| ***Use of visuals during conversation (videos, pictures, drawings, flyers, etc.).*** |  |
| ***Time for client to ask questions on PrEP and how to use it.*** |  |
| ***Counseling on PrEP adherence.*** |  |
| ***Counseling on sexual health and how to achieve it.*** |  |
| ***Discussion on vaccination.*** |  |
| ***Exploration of other needs of the client.*** |  |
| ***Delivery of information on HIV and STI testing.*** |  |
| ***Discussion on how follow-up for PrEP is organized.*** |  |
| ***Collaboration with other health professionals (including the client’s GP).*** |  |
| ***Remaining questions.*** |  |

- ***Verbal communication of the provider***
  - ***Pace (too fast?)***
  - ***Volume (too loud, too silent?)***
  - ***Tone (respectful, non-judgmental)***
  - ***Language (Understandable? Inclusive? Neutral? Excessive use of jargon?)***
  - ***Dosage (not too much too quickly)***
  - ***Patient-centered (responsive to needs of the client, reassures client, asks opinion of the client regularly, etc.)***
- ***Verbal communication of the client***
  - ***Talkative? Or rather silent?***
  - ***Amount of questions (lots of questions?)***
  - ***Nature of questions (reflects a good or rather poor knowledge base on health-related issues?)***
  - ***Tone (shy vs. assertive/confident)***
  - ***Are questions answered?***
- ***Non-verbal communication of the provider***
  - ***Attitude (warm & welcoming vs. closed & distant)***
  - ***Eye contact (looking at screen, papers, busy writing instead of addressing client?)***
  - ***Signs of active listening (nodding, humming, smiling,…)***
  - ***Keeps time management (‘hurried’ impression, looking at the clock,…)***
- ***Non-verbal communication of the client***
  - ***Attitude (receptive, spontaneous vs. closed, not much feedback)***
  - ***Facial expression (neutral, confused, worried,…)***
  - ***Overall impression (nervous, relaxed, patient, in a hurry,…)***

1. **CLIENT FLOWS**

***Aim is to get a clear picture of how clients ‘move’ through the PrEP clinic, which different ‘routes’ are possible, and to have an idea of how much time – on average – that takes them.***

| ***Schematic overview of how clients can move through different stages of their PrEP visit*** |
| --- |

- ***How do PrEP clients enter the clinic? (different options – entrances?)***
- ***Where do PrEP clients register? (different options – locations?)***
- ***Where do PrEP clients move after registration? (different options – locations/waiting areas?)***
- ***Which provider do PrEP clients first encounter?***
  - ***Are there different possibilities (e.g. according to type of visit: screening versus follow-up, or according to availability of provider, etc.)***
- ***Which other providers do PrEP clients encounter, and in which order?***
  - ***Are there different possibilities (e.g. according to type of visit: screening versus follow-up, or according to availability of provider, etc.)***
- ***Are clients in-between stages of the PrEP visit sometimes referred back to the waiting room?***
  - ***If yes, for what reasons?***
  - ***For how long (on average)?***
- ***At what stage during the visit can clients receive a STI/HIV test?***
  - ***Does this happen during the PrEP visit?***
  - ***Where do they go for this?***
  - ***Can they do the sampling themselves?***
  - ***Is there a waiting line for this?***
- ***Who is the last provider clients encounter before they exit?***
  - ***Does this sometimes differ? If yes, according to what?***
- ***Where do clients exit? (different options – exits?)***
- ***Time log below can be used to have an idea about durations (on average) – to be adapted based on information from key informants about specific client flows in different HRCs***

| ***Time log (try to complete for a few clients, to have an idea of time management)*** | |
| --- | --- |
| **Date:** | |
| **STAGE 1**  **…………………………………**  **(*location & provider)*** | ***Time enter:***  ***Time exit:*** |
| **STAGE 2**  **…………………………………** | ***Time enter:***  ***Time exit:*** |
| **STAGE 3**  **…………………………………** | ***Time enter:***  ***Time exit:*** |
| **STAGE 4**  **…………………………………** | ***Time enter:***  ***Time exit:*** |
| **STAGE 5**  **…………………………………** | ***Time enter:***  ***Time exit:*** |
| **STAGE 6**  **…………………………………** | ***Time enter:***  ***Time exit:*** |
| **STAGE X**  **………………………………….** | ***Time enter:***  ***Time exit:*** |

| ***Field notes and general summary of the observation*** |
| --- |
