# Supplementary material for: Understanding adaptive responses in PrEP service delivery in Belgian HIV clinics: a multiple case study using an implementation science framework
Source: J Int AIDS Soc. 2024 Jul 5;27(Suppl 1):e26260. doi: 10.1002/jia2.26260 (PMC11224588; doi:10.1002/jia2.26260)
Supplement: Supplementary file 4 — File S4: Extended Normalisation Process Theory constructs, definitions, themes and sub‐themes derived from the data [file JIA2-27-e26260-s003.docx]

**Understanding adaptive responses in PrEP service delivery in Belgian HIV clinics: a multiple case study using an implementation science framework**

**Supplementary file 4.**

**Extended Normalisation Process Theory constructs, definitions and themes and sub-themes derived from the data.**

**Supplementary file 4. Extended Normalisation Process Theory (eNPT) constructs, definitions and thematic coding framework applied to the data.**

| **Concept** | **eNPT construct** | **Definition** | **Thematic coding framework (i.e. themes and subthemes)** |
| --- | --- | --- | --- |
| Expressions of adaptive change | Normative restructuring | Modifications to the conventions, rules and resources that participants in the intervention or programme experience as providing the scaffolding for everyday behaviour and action. | 1. *Re-organising clinic structures (Theme 1)* 2. Reactive rather than proactive 3. Efficiency-driven 4. Practical strategies    1. Engaging additional staff    2. Protocolising workflows 5. Organisational strategies    1. Integration with other services    2. Establishing separate “PrEP clinics” 6. Compensatory limitations    1. Unmet PrEP demand    2. Waiting times for new clients    3. Laboratory capacity limits 7. *Progressing clinical practice norms (Theme 2).* 8. Efficiency-driven    1. Reducing visit frequency 9. Meeting clients’ needs    1. Convenience    2. Psychosocial needs    3. PrEP need 10. Tension with rules and regulations     1. PrEP eligibility     2. PrEP follow-up practices 11. Reducing barriers for clients     1. Alternative visit frequency     2. Alternative locations     3. Alternative opening hours     4. Alternative care providers |
|  | Relational restructuring | Changes to the ways in which participants in the intervention or programme are organised and relate to each other. | *1. Expanding roles and responsibilities for nurses (Theme 1).*   1. Nurses as PrEP experts    1. Degree of autonomy of nurses    2. Role of clinic managers and leadership    3. Facilitating environment    4. Professional identity and confidence 2. Nurses as caregivers    1. Responding to clients’ non-medical needs    2. Role of time management    3. Filling gaps left by physicians    4. Flexibility and tailoring capacity   *2. Engaging with psychosocial expertise (Theme 2).*   1. Responding to clients’ psychosocial needs    1. Sexualised drug use    2. Compulsive sexual behaviour    3. HIV anxiety    4. Mental health 2. Available psychosocial expertise    1. Programme funding    2. Historical involvement of psychologists in HIV care    3. On-site expertise 3. Strategies    1. Incorporating in PrEP pathway    2. Demand-driven referrals    3. Re-training of nurses 4. *Relations and interactions with family physicians (Theme 3).* 5. Active vs. passive    1. Role of clinic leadership    2. Clinic workload    3. Trust in FPs’ capacity and potential    4. Tensions with rules and regulations    5. Ideas on professional expertise |
| Dynamic elements of context | Capacity | The extent to which material, cognitive and social resources are available to individuals and organisations tasked with operationalising the intervention or programme. | 1. *HIV and sexual health expertise (Theme 1).* 2. Historical expertise of HIV clinics 3. Degree of specialisation 4. Skills to respond to needs of sexual minorities 5. *Access to professional networks (Theme 2).* 6. Historical networks 7. Conferences 8. Literature 9. Formal and informal networks with specialists 10. *Clinic resources in relation to PrEP demand (Theme 3).* 11. *Co-location of different providers (Theme 4).* 12. *Available funding for HIV and PrEP programmes (Theme 5).* 13. *Connections and collaborations with primary care (Theme 6).* |
|  | Potential | Readiness to operationalise the intervention; includes the factors that shape the individual and collective motivation and commitment among participants to engage with the (new) intervention or programme. | 1. *Alignment of PrEP with organisational mission and goals (Theme 1).* 2. *Clinic managers’ commitment to PrEP care (Theme 2).* 3. *Staff motivation and interest in PrEP (Theme 3).* 4. *Open communication and trust across professional cadres (Theme 4).* 5. *Teamwork and shared goals (Theme 5).* 6. *Attitudes towards interprofessional collaboration (Theme 6).* |
