# Supplementary material for: Understanding adaptive responses in PrEP service delivery in Belgian HIV clinics: a multiple case study using an implementation science framework
Source: J Int AIDS Soc. 2024 Jul 5;27(Suppl 1):e26260. doi: 10.1002/jia2.26260 (PMC11224588; doi:10.1002/jia2.26260)
Supplement: Supplementary file 5 — File S5: Detailed overview of the variation in PrEP service delivery across the cases [file JIA2-27-e26260-s002.docx]

**Understanding adaptive responses in PrEP service delivery in Belgian HIV clinics: a multiple case study using an implementation science framework**

**Supporting information file 5.**

**Illustrations of observed variation in implementation of some key characteristics of PrEP service delivery across the cases.**

| 1. **PrEP clinic structure** | |
| --- | --- |
| ***General observation:*** While HIV clinics share similar responsibilities and the same administrative mandate, there were important (historical) differences across cases in terms of infrastructure and organisational structure already before the time of PrEP implementation. These differences co-determined the variation in how clinic structures were re-organised to ensure a contextual and logistical fit with PrEP delivery. An important driver of these changes was the felt need to adapt based on the increased workload of PrEP over time, reflecting local differences in PrEP demand in relation to clinic resources. | |
| Service delivery characteristic | Variation in implementation across cases |
| Location of PrEP service delivery | - In five cases (C,D,F,G and H), PrEP services were delivered in an out-patient unit of a larger hospital setting wherein the HIV clinic was integrated. Staff and infrastructure were often shared for other out-patient services offered at these hospital units (e.g. internal medicine, infectious diseases and/or travel medicine). - In two cases (A and B), PrEP services were accommodated in pre-existing (non-hospital based) urban sexual health clinics that were separated from the affiliated hospital services for in-patient care. These settings historically offered more preventive sexual health services, including low-threshold and anonymous STI testing and treatment options within the community, and were deemed a better fit with the preventive purpose of PrEP than in-hospital services. - In one case (E), PrEP services were integrated in a policlinic offering multiple out-patient services associated with, but separated from, the larger hospital environment wherein the HIV clinic (and other in-patient care) was embedded. A separate urban satellite clinic offered PrEP services to people experiencing difficulties traveling to the main policlinic (located outside the city centre). |
| Level of integration of PrEP with other services at the HIV clinic | - In three cases (B, E and F) PrEP visits were combined with other types of visits offered at the same time (e.g. HIV and STI care, and clients coming for PEP), with the same care providers attending to all clients. - In five cases (A,C,D,G and H), dedicated ‘PrEP clinics’ were organised at particular time slots, with care providers only seeing clients coming in for PrEP on those time periods. |
| Appointment system for PrEP visits | - One case (A) offered clients the option to book their own visits online, whereas the other cases only worked with appointments by telephone. - One case (B) offered a walk-in option for PrEP visits (i.e. no appointment needed) during a particular time of the week. - Available time slots for PrEP visits varied between two days per month (case D), one day per week (case C and G), 2-4 days per week (case A,B, F and H) and every day of the week (case E). |
| 1. **PrEP care practices** | |
| ***General observation:*** The variation in PrEP care practices across cases could be explained by the lack of a uniform clinical standard (i.e. national PrEP guidelines) endorsed by all HIV clinics. In addition, PrEP practice norms progressed within cases over time – at different speeds – based on evolving clinical experience with PrEP care and pushed by increasing workload of a growing PrEP client cohort. This led to gradually enacting more differentiated PrEP care approaches, at times extending or overriding policy-commissioned regulations on PrEP care, that had the potential to meet individual client needs while reducing pressure on busy HIV clinics. | |
| Service delivery characteristic | Variation in implementation across cases |
| PrEP initiation procedures | - Four cases (A,B,E and F) had a step-wise approach towards initiating PrEP, requiring at least two facility visits to deliver the first PrEP prescription: at a first screening visit the necessary laboratory tests were done, and at a separate visit (about 1-2 weeks later) test results were checked and a PrEP prescription was delivered. The other four cases (C,D,G and H) allowed for ‘same-day PrEP initiation’, conducting the necessary laboratory screening and delivering the first PrEP prescription on the same day in case of no acute HIV symptoms, with the instruction to start PrEP after a clinician had checked and notified clients (i.e. by telephone) of a required negative HIV test result. - Three cases (E,F and G) conducted routine one-month facility visits after PrEP initiation to assess adherence and PrEP-related toxicity or side effects, while the other cases had removed those visits over time. - In three cases (C,E and F) a first PrEP prescription could be filled in the hospital pharmacy immediately after the PrEP visit. In the other cases, PrEP prescriptions needed to be filled at community pharmacists outside the clinic/hospital. |
| PrEP follow-up routines | - Two cases (B and E) required two facility visits per PrEP follow-up contact: one to conduct the necessary laboratory testing, followed by a visit about one week after to discuss test results and provide a refill prescription of PrEP. The other cases (A,C,D,F,G and H) combined laboratory testing with delivering a PrEP refill prescription at the same visit, combined with a system no notify clients about their test results after the visit (e.g. by telephone or e-mail). - All but one case (A,B,C,D,E,F and G) offered facility-based self-sampling for STI screening during PrEP follow-up visits. - One case (A) deviated more systematically from the quarterly visit policy for PrEP follow-up, offering on-demand PrEP users with low HIV risk the option of 4-monthly up to 6-monthly visit frequencies (depending on PrEP use and sexual behaviour). In other cases, such deviations were less frequent, mainly based on explicit client requests. |
| 1. **PrEP care professionals** | |
| ***General observation:*** Differences in staffing across the cases determined which provider profiles were more likely to be early PrEP providers. These were initially predominantly specialised infectious disease physicians and/or general practitioners with historical expertise in HIV or sexual health. Engaging additional staff (incl. nurses, psychologists/sexologists and family physicians) in PrEP care depended on their availability, their existing function in the HIV clinic and on clinic leaders’ attitudes and initiatives towards proactively involving other care providers in the PrEP workflow. | |
| Service delivery characteristic | Variation in implementation across cases |
| Degree of task-shifting/task-sharing between physicians and nurses | - In one case (B), the sexual health clinic (incl. PrEP care) was mainly staffed by general practitioners with a special interest in sexual health from the communities around the clinic. Task-shifting to nurses was limited, with nurses mainly performing technical acts (e.g. taking blood samples and administering injections). - In two cases (A and C), time-intensive counselling and education tasks were shifted to nurses, with physicians attending to all PrEP clients (i.e. initiation and follow-up) to discuss/order laboratory testing and prescribe PrEP. - In five cases (D,E,F,G and H), PrEP visits were shared between physicians and nurses, with nurses having the possibility to handle routine PrEP visits semi-autonomously (i.e. with remote physician oversight and/or only relying on physicians to order PrEP prescriptions from a distance). |
| Collaboration between PrEP clinicians and on-site sexologists/psychologists | - In one case (B), a sexologist/psychologist was co-located with other PrEP clinicians, with immediate availability during PrEP consultations for on-demand referrals (i.e. based on judgment of PrEP clinician). - In four cases (A,C,D and H) a sexologist/psychologist was available for on-demand referrals through a separate appointment (i.e. about 1-2 weeks later) made by the referring PrEP clinician. - In two cases (E and F) some of the nurses routinely conducting PrEP visits had a dual degree in sexology, combining PrEP visits with immediate specialised support where needed. - In one case (G) a psychologist/sexologist was structurally incorporated in the PrEP client flow, committed to seeing all PrEP clients around the time of PrEP initiation. |
| Involvement of clients’ family physician in PrEP care | - Four cases (A,B,E and F) reported very little interaction with PrEP clients’ family physician, and did not routinely communicate/report on PrEP visits to clients’ family physician. - Three cases (C,D and G) reported sending routine reports about PrEP visits to clients’ family physician, but did not actively involve them in PrEP care aspects. - One case (H) reported sending routine reports about PrEP visits to clients’ family physician and proactively offered all PrEP clients the option of alternating follow-up visits at the HIV clinic with visits at their family physician. |
